# Plasma IL-37 Elevated in Patients with Chronic Heart Failure and Predicted Major Adverse Cardiac Events: A 1-Year Follow-Up Study

**DOI:** 10.1155/2017/9134079

**Published:** 2017-07-11

**Authors:** Xiling Shou, Jing Lin, Cui Xie, Yi Wang, Chaofeng Sun

**Affiliations:** ^1^Department of Cardiology, The First Affiliated Hospital Medical College of Xi'an Jiaotong University, Xi'an 710061, China; ^2^Department of Cardiology, Shaanxi Province People's Hospital and The Third Affiliated Hospital Medical College of Xi'an Jiaotong University, Xi'an 710068, China

## Abstract

A great number of basic and clinical studies have demonstrated that inflammatory cytokines play an important role in the development and progression of chronic heart failure (CHF). However, there is limited information about the role of novel cytokine interleukin-37 (IL-37) in heart failure. We measured plasma IL-37 levels by enzyme-linked immunosorbent assay (ELISA) in 158 patients with chronic heart failure and 30 control subjects. Our results showed that plasma IL-37 levels were significantly elevated in patients with CHF compared with healthy controls (143.73 ± 26.83 pg/ml versus 45.2 ± 11.56 pg/ml, *P* < 0.001). Furthermore, plasma IL-37 levels were positively correlated with hs-CRP, hs-TnT, and NT-proBNP and negatively correlated with left ventricular ejection function (LVEF). 11 patients died of cardiovascular cause, and 27 HF patients were rehospitalized for worsening HF within 12 months. Multivariate Cox regression analysis showed that plasma IL-37 is an independent predictor of major adverse cardiac events (MACE). Furthermore, CHF patients with >99 pg/ml plasma IL-37 had significantly higher incidences of MACE within 12 months. Our data suggest that plasma IL-37 may play a role in the pathogenesis of CHF and may be a novel predictor of poor prognosis in HF patients.

## 1. Introduction

Chronic heart failure (CHF) is defined as impaired cardiac structure and/or function in ventricular filling or ejection that result in a complex set of clinical syndromes [1]. With the development of immunohistochemistry and molecular cell biology, the basic mechanism of heart failure has been proven to be due to cardiac remodeling characterized by necrosis and apoptosis of cardiomyocytes and progressive expansion of the ventricular cavity [2, 3].

A large number of studies have shown a close association between inflammation and cardiac remodeling. Inflammatory mediators, especially tumor necrosis factor- (TNF-) *α*, interleukin- (IL-) 6, IL-1*β*, and IL-18, impair cardiac function by promoting cardiomyocyte apoptosis, cardiac hypertrophy, inflammatory response, and matrix metalloproteinase-9 (MMP-9) activity; their plasma levels are increased in heart failure in association with disease severity [3–5]. Many groups therefore proposed inhibiting inflammation as a potent therapeutic target in heart failure [3, 5]. However, CHF is a more complicated process and there remain many unknowns regarding the relationship between inflammation and heart failure.

Interleukin-37 belongs to the IL-1 ligand family and is a newly identified anti-inflammatory cytokine [6]. IL-37 inhibits the secretion of proinflammatory cytokines such as IL-1*β*, IL-6, and TNF-*α* in peripheral blood monocytes, macrophages, dendritic cells, and epithelial cells, playing a critical role in innate immunity and adaptive immunity [7]. A great number of studies demonstrated that IL-37 is involved in the occurrence and development of chronic inflammation and autoimmune diseases such as rheumatoid arthritis, systemic lupus erythematosus, and diabetes [8–11]. In addition, evidence from clinical and animal studies has confirmed that IL-37 not only participates in atherosclerotic disease but also has a close relationship with impaired heart function [12–14]. However, the level of plasma IL-37 in heart failure has yet been investigated. The goal of our study was to examine the plasma IL-37 level in patients with CHF and assess its relation to clinical parameters and biochemical laboratory data.

## 2. Materials and Methods

### 2.1. Study Population

A total of 158 patients were enrolled in this study. The diagnosis of CHF was based on typical symptoms and signs of heart failure and evidence of left ventricular enlargement and systolic functional impairment on echocardiography, according to the American College of Cardiology/American Heart Association guidelines [1]. Exclusion include patients with CHF secondary to specific aetiologies (e.g., malignant disease, chronic inflammatory disease, and/or infiltrative or congenital heart disease) and with end-stage renal failure (defined as estimated glomerular filtration rate *<* 15 ml/min·m^2^). Thirty healthy individuals were selected as control subjects who matched with CHF patients in age, gender, and body mass index (BMI).

Every participant provided written informed consent, and the study was approved by the hospital ethical review board (Shaanxi Province People's Hospital and Center for Cardiovascular Diseases, China). All study procedures were in accordance with the ethical standards outlined in the Declaration of Helsinki of 1975, as revised in 1983.

### 2.2. Study Procedures

Once recruited, baseline assessments involve standardized history taking, physical examination, a resting 12-lead electrocardiogram, chest X-ray, blood sampling, and comprehensive transthoracic Doppler echocardiography using standardized equipment (Vivid ultrasound systems, General Electric, Milwaukee, WI, USA) complying with recommendations from the American Society of Echocardiography (2009). Coronary angiography was performed to define ischemic heart disease (IHD) and non-IHD as needed. All patients were followed up to 12 months from discharge to evaluate major adverse cardiac events (MACE), which was defined as first rehospitalization for CHF or death due to cardiovascular cause.

### 2.3. ELISA Detection of the Levels of Plasma IL-37

The level of plasma IL-37 (Adipogen AG, Liestal, Switzerland) was measured by an enzyme-linked immunosorbent assay (ELISA), following the manufacturer's instructions. The minimal detectable concentration of IL-37 by this assay is 10 pg/ml for IL-37. The ELISA intra-assay and inter-assay coefficients of variation are <5% and <10%, respectively. All of the samples were measured in duplicate.

### 2.4. Statistical Analysis

The SPSS 17.0 software package (SPSS, Chicago, IL, USA) was employed for statistical processing. Measurement data were presented as mean ± SD or median. Numeration data were presented as a constituent ratio. All continuous variables were tested for normal distribution and homogeneity for variance. Comparisons of CHF patients versus control subjects and the subgroup of CHF patients were performed using the two-tailed Student *t*-test. Coefficients of correlation (*r*) were calculated using Pearson's correlation coefficient. Hazard ratios (HR) and 95% confidence intervals (CI) were calculated for each factor with Cox proportional hazards analysis. To identify independent predictors of major adverse cardiac events, all baseline variables with *P* < 0.05 in the univariate analysis were entered into a multivariate model. In addition, differences in event-free survival by median of plasma IL-37 (IL-37 ≤ 99 pg/ml and IL-37 > 99 pg/ml) were examined using the Kaplan-Meier method and compared using a log-rank test. Differences were considered statistically significant at *P* < 0.05.

## 3. Results

### 3.1. Clinical Characteristics in Patients with CHF

The baseline clinical characteristics of patients with CHF are summarized in Table 1. The mean age of patients was 65.25 ± 9.63 years, and 67.72% were male. The mean BMI of patients was 23.33 ± 2.09 kg/m^2^. The proportion of patients with a diagnosis of ischemic heart disease (IHD), hypertension (HP), and diabetes mellitus (DM) was 56.33%, 46.20%, and 18.35%, respectively. The distribution of patients among the New York Heart Association (NYHA) cardiac function class included 62.03% from class II/III and 37.97% from class IV. The proportion of patients who were taking drugs was 88.61% of an angiotensin-converting enzyme inhibitor (ACEI)/angiotensin II receptor blocker (ARB), 83.54% of beta-blocker, 74.68% of loop diuretic, 44.30% of aldosterone antagonist, and 28.48% of digoxin. The mean left ventricular ejection function (LVEF) of CHF patients was 37.82 ± 4.90%. The concentration of plasma biomarkers hs-TnT, hs-CRP, and NT-proBNP was 37.82 ± 4.90 pg/ml, 3.62 ± 1.08 ng/ml, and 2043.59 ± 1094.89 pg/ml, respectively. In this study, the median length of follow-up was 109 days (range 35 to 365 days). No patient was lost to the follow-up.

### 3.2. Plasma IL-37 Elevated in CHF Patients

**Figure 1:**
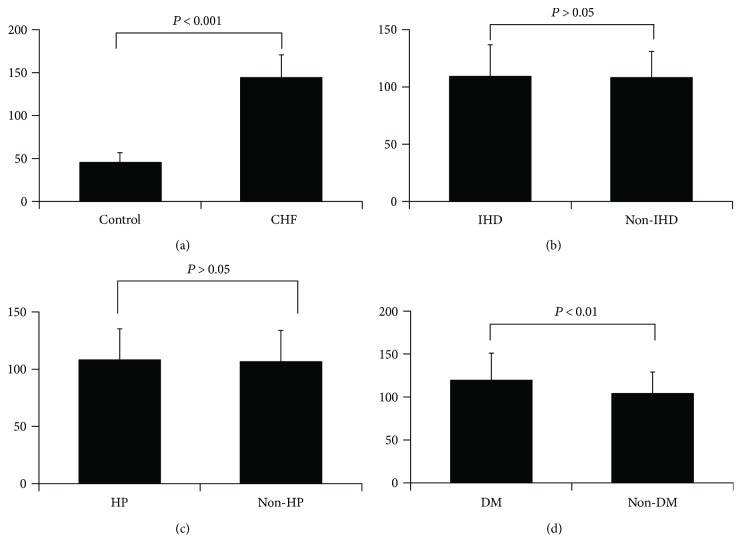
Elevated plasma IL-37 in chronic heart failure. (a) Plasma IL-37 in chronic heart failure (CHF) patients compared with control subjects (control); (b) plasma IL-37 levels in ischemic heart disease (IHD) subgroup and non-IHD subgroup; (c) plasma IL-37 levels in hypertension (HP) subgroup and nonhypertension subgroup (non-HP); (d) plasma IL-37 levels in diabetes mellitus (DM) subgroup and non-DM subgroup (non-DM).

The mean plasma IL-37 level in patients with CHF was significantly elevated (143.73 ± 26.83 pg/ml) compared with that in control subjects (45.2 ± 11.56 pg/ml) (*P* < 0.001) (Figure 1(a)). In subgroup analyses of CHF patients, there was no significant difference between patients with IHD and without IHD, as well as patients with hypertension (HP) and with normal blood pressure (non-HP) (all *P* > 0.05) (Figures 1(b) and 1(c)). However, plasma IL-37 level in CHF patients with DM was significantly higher compared to that in patients without diabetes (*P* < 0.01) (Figure 1(d)).

### 3.3. Correlation of Plasma IL-37 with LVEF and Biomarkers

**Figure 2:**
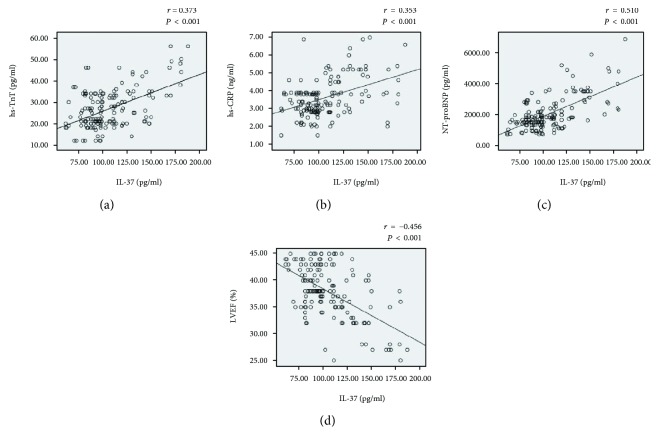
Correlation between plasma IL-37 and LVEF and biomarkers. (a), (b), (c) plasma IL-37 positively correlates with hs-TnT, hs-CRP, or NT-proBNP; (d) plasma IL-37 negatively correlates with LVEF.

Next, we examined the correlation between plasma IL-37 and biomarkers of cardiac events, disease, and function and LVEF. As shown in Figure 2, plasma IL-37 positively correlated with hs-TnT (Figure 2(a)), hs-CRP (Figure 2(b)), and NT-proBNP (Figure 2(c)) (all *P* < 0.001). However, plasma IL-37 negatively correlated with LVEF (Figure 2(d)) (*P* < 0.001).

### 3.4. Higher Plasma IL-37 Is an Independent Predictor for MACE within 12 Months in CHF Patients

In this study, 38 major adverse cardiac events of 158 CHF patients were recorded within 12 months from discharge, including 11 patient deaths and 27 patient rehospitalizations for worsening HF. In the univariate Cox regression model, BMI, DM, LVEF, hs-TnT, hs-CRP, NT-proBNP, and IL-37 were associated with MACE in CHF patients (Table 2). When we performed Cox stepwise multivariate analysis including all variables with *P* < 0.05 on a univariate analysis, plasma IL-37 and NT-proBNP were significant predictors of MACE within 12 months of follow-up.

**Figure 3:**
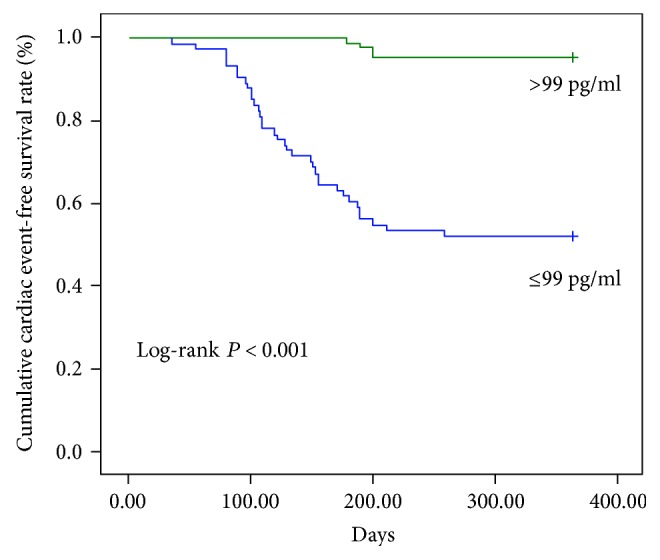
Kaplan-Meier curves demonstrating MACE in CHF patients during 12 months from discharge. Green line: CHF patients with lower concentration of plasma IL-37 (≤99 pg/ml); blue line: CHF patients with higher concentration of plasma IL-37 (>99 pg/ml). Log-rank test, *P* < 0.001.

To determine the predictive value of the concentration of IL-37 on MACE, we divided CHF patients into a subgroup with IL-37 ≤ 99 pg/ml and >99 pg/ml by median levels of plasma IL-37. Kaplan-Meier curves and log-rank testing revealed that CHF patients with a higher concentration of plasma IL-37 (>99 pg/ml) had significantly higher MACE within 12 months from discharge (Figure 3) (*P* < 0.001).

## 4. Discussion

In the present study, our results showed that plasma IL-37 levels were significantly elevated in patients with CHF compared with healthy controls (143.73 ± 26.83 pg/ml versus 45.2 ± 11.56 pg/ml, *P* < 0.001). Furthermore, plasma IL-37 levels positively correlated with hs-CRP, hs-TnT, and NT-proBNP and negatively correlated with LVEF. 11 patients died of cardiovascular cause, and 27 patients were rehospitalized for worsening HF within 12 months. Multivariate Cox regression analysis showed that plasma IL-37 is an independent predictor of MACE in patients with CHF. Furthermore, CHF patients with >99 pg/ml plasma IL-37 had significantly higher incidences of MACE within 12 months. Our data suggest that plasma IL-37 might be involved in the pathogenesis of CHF and may be a novel predictor of poor prognosis in HF patients.

IL-37 is a novel homolog of the IL-1 cytokine family discovered by computational cloning and was originally designated as IL-1H4 in 2000 [15]. IL-37 is synthesized as a precursor molecule that needs to be cleaved by caspase-1 to generate mature IL-37 [16]. The production of IL-37 occurs at low levels in a physiological state and can be effectively induced in an inflammatory environment. Studies have shown that inflammatory stimulants such as IFN-*γ*, TNF-*α*, and lipopolysaccharide (LPS) promote the expression of IL-37 by peripheral blood mononuclear cells, dendritic cells, and epidermal cells. Recent studies confirmed that activated T lymphocytes also secrete IL-37 in an inducible manner [17]. Both endogenous and exogenous IL-37 have been shown to ameliorate inflammation and regulate immune disorder via inhibiting the production of inflammatory mediators including IFN-*γ*, TNF-*α*, IL-6, and IL-18 [6, 7].

Accumulating evidence shows that IL-37 plays a critical role in cardiovascular disease [7, 12–14]. Boraschi et al. first found high expression of IL-37 in atherosclerotic coronary and carotid artery plaques [7]. Ji et al. observed that circulating IL-37 levels are significantly increased and correlated with inflammatory markers and impaired left ventricular function in patients with acute coronary syndrome [12]. By using a myocardial ischemia/reperfusion injury model, Wu et al. found that exogenous IL-37 reduced infarct size, decreased cardiac troponin T levels, and improved cardiac function via suppressing the production of proinflammatory cytokines and chemokines and the infiltration of leukocyte [13]. Another study showed that IL-37 treatment can improve cardiac function through inhibiting the activation of NF-*κ*B signaling pathway in a myocardial infarction model [14]. Coronary artery disease/myocardial infarction is one of the important causes of heart failure. In the present study, we are the first to demonstrate that plasma IL-37 becomes elevated in chronic heart failure patients, indicating a potential role of IL-37 in the development of heart failure.

Cardiac remodeling is the fundamental pathological process of heart failure. It is defined as structural and functional changes in the myocardium that result in left ventricular dilatation leading to heart failure [18]. In this study, we found that baseline IL-37 levels in patients with heart failure negatively correlate with LVEF while positively correlating with NT-proBNP and hs-TnT, the two most extensively studied biomarkers in evaluating the severity of cardiac function. These results are partly consistent with previous research that showed plasma IL-37 levels correlate with inflammatory markers and impaired left ventricular function in patients with acute coronary syndrome [12]. However, it is noted that our results found no significant differences in plasma IL-37 levels between the IHD and non-IHD subgroups. This observation seems contradictory with previous findings of elevated plasma IL-37 levels in patients with acute coronary syndrome. Because numerous research studies have confirmed the role of inflammation in acute coronary syndrome, the possible explanation for this contradiction is that IL-37 levels in plasma may correlate with the grade of inflammation rather than the disease status. This notion is further solidified by our results that plasma IL-37 levels positively correlate with hs-CRP, a well-known biomarker of inflammation [19]. Also, we did not observe significant differences in plasma IL-37 levels in the HP and non-HP subgroups. Interestingly, our study shows significantly increased IL-37 levels in the DM subgroup compared to that in the non-DM subgroup. Although Ballak et al. found that elevated IL-37 levels positively correlate with insulin sensitivity and a lower inflammatory status in human adipose tissue and that IL-37 ameliorates obesity-induced inflammation and insulin resistance in transgenic mice, the reasonable possibility for this discrepancy is that the DM status more closely correlates with the poor cardiac function in CHF patients [10, 20].

Finally, in this study, we demonstrated for the first time that plasma IL-37 is an independent predictor for MACE within 12 months. There are numerous reports that NT-proBNP and LVEF are predictors of poor outcomes in heart failure patients [21, 22]. Impaired LVEF is a hallmark of heart failure and reflects a fundamental weakness of the pump. It is no doubt that the lower the LVEF, the worse the prognosis of patients with heart failure. In this study, we showed that the higher concentration of plasma IL-37 (>99 pg/ml) is a significant predictor of recurrent hospitalizations for worsening HF and deaths due to a cardiovascular cause, independent of other clinical and laboratory variables. We believe that the increase in plasma IL-37 is associated with inflammation, cardiac remodeling, and acute cardiovascular events in heart failure patients. However, a direct causal relationship between an increase in plasma IL-37 and inflammation was not found in the study.

In conclusion, our study is the first to investigate the association between plasma IL-37 levels and chronic heart failure. We found that plasma IL-37 levels are significantly increased in patients with CHF and that the increase in plasma IL-37 levels is an unfavorable prognosis for patients with heart failure. However, there are some limitations in the present study. When performing the prognostic analysis, a sample population of 158 subjects is too small and a 12-month follow-up is too short, although statistical significance has been found. Therefore, a prospective trial consisting of a larger number of patients with heart failure and longer period of follow-up needs to be performed to clarify the significance of circulating IL-37 levels in heart failure. Furthermore, to fully assess the role of plasma IL-37 on cardiac inflammation and left ventricular remodeling, future studies are required to examine the underlying mechanism responsible for the increase of plasma IL-37 in patients with chronic heart failure.

## Figures and Tables

**Table 1:** Baseline characteristics of patients with chronic heart failure.

Variables	CHF (*n* = 158)
Age (years)	65.25 ± 9.63
Male, *n* (%)	107 (67.72)
BMI (kg/m^2^)	23.33 ± 2.09
IHD, *n* (%)	89 (56.33)
Hypertension, *n* (%)	73 (46.20)
DM, *n* (%)	29 (18.35)
NYHA class, *n* (%)	
II/III	98 (62.03)
IV	60 (37.97)
Medication, *n* (%)	
ACE-I/ARB	140 (88.61)
Beta-blocker	132 (83.54)
Loop diuretic	118 (74.68)
Aldosterone antagonist	70 (44.30)
Digoxin	45 (28.48)
LVEF (%)	37.82 ± 4.90
hs-TnT (pg/ml)	26.86 ± 9.19
hs-CRP (ng/ml)	3.62 ± 1.08
NT-proBNP (pg/ml)	2043.59 ± 1094.89

Values are mean ± standard deviation or proportions.

**Table 2:** Cox regression analysis for major adverse cardiac events.

Variables	Univariate analysis		Multivariable analysis	
	Hazard ratio		Hazard ratio	
	(95% CI)	*P* value	(95% CI)	*P* value
Age (years)	0.974 (0.944–1.005)	0.098		
Male, *n* (%)	0.647 (0.342–1.225)	0.181		
BMI (kg/m^2^)	1.179 (1.010–1.376)	0.037	1.301 (1.042–1.623)	0.020
IHD, *n* (%)	1.489 (0.774–2.865)	0.233		
Hypertension, *n* (%)	1.272 (0.679–2.384)	0.452		
DM, *n* (%)	2.797 (1.435–5452)	0.003	3.077 (1.435–5.452)	0.027
LVEF (%)	0.800 (0.749–0.854)	<0.001	0.925 (0.848–1.009)	0.077
hs-TnT (pg/ml)	1.097 (1.060–1.136)	<0.001		
hs-CRP (ng/ml)	1.982 (1.539–2.553)	<0.001		
NT-proBNP (pg/ml)	1.001 (1.001–1.001)	<0.001	1.001 (1.000–1.001)	<0.001
IL-37 (pg/ml)	1.053 (1.043–1.064)	<0.001	1.038 (1.017–1.059)	<0.001
